# Colchicine Myopathy: A Case Series Including Muscle MRI and *ABCB1* Polymorphism Data

**DOI:** 10.3389/fneur.2019.00553

**Published:** 2019-05-24

**Authors:** Mehul Gupta, Ana Nikolic, Denise Ng, Kristina Martens, Hamid Ebadi, Sameer Chhibber, Gerald Pfeffer

**Affiliations:** ^1^Hotchkiss Brain Institute, University of Calgary, Calgary, AB, Canada; ^2^Department of Pathology and Laboratory Medicine, University of Calgary, Calgary, AB, Canada; ^3^Department of Clinical Neurosciences, Cumming School of Medicine, University of Calgary, Calgary, AB, Canada

**Keywords:** colchicine, statin, myopathy, vacuolar, muscle MRI, pharmacogenetics

## Abstract

Colchicine is a medication most commonly used in the treatment of gout and familial mediterannean fever. A rare complication of therapy is toxicity causing proximal myopathy and polyneuropathy. Colchicine myopathy has been associated with the coadministration of other medications with colchicine, such as statins or tacrolimus, and is more common in patients with renal impairment. Otherwise, it is unclear which patients are at greatest risk of developing this adverse drug reaction. *ABCB1* is important to the metabolism of colchicine, so we speculated that it was possible that colchicine myopathy patients may have a particular genotype that is associated with this side effect. We describe two cases of colchicine myopathy which occurred with co-administration of rosuvastatin. From one case, we present the first published data on muscle MRI in this condition. We additionally present an analysis of four genetic polymorphisms in *ABCB1* and transcript levels in muscle tissue, and demonstrate the descriptive finding of reduced *ABCB1* transcript levels in the colchicine myopathy patients.

## Introduction

Colchicine-induced myopathy is an autophagic, vacuolar myopathy that occurs as a rare complication of treatment with colchicine (1). The development of colchicine myopathy has been predominantly reported in patients using colchicine in the context of renal failure (1), although it has been associated with other medications such as statins (2), or tacrolimus (3). The clinical presentation typically includes features of a painless proximal myopathy, that develops slowly after prolonged treatment with colchicine (4), but may also develop precipitously with rhabdomyolysis (5). The onset can occur within weeks of initiating colchicine therapy, particularly in those with renal impairment (6). Significant variability can occur in the clinical presentation (7), and colchicine myopathy has also been reported with respiratory muscle weakness (8), making this rare medication side-effect a consideration in a broad range of neuromuscular differential diagnoses (9, 10). The diagnosis is typically confirmed with the finding of a vacuolar myopathy on muscle biopsy (11). Some features of this condition, such as muscle MRI findings, are currently unknown.

One of the important factors in colchicine's metabolism is *ABCB1* (also known as multidrug resistance protein 1, or p-glycoprotein, encoded by *ABCB1*) (12). There is some indication that genetic variation in *ABCB1* may contribute to the variability in colchicine's effectiveness for familial Mediterranean fever (13–15), though the literature appears to be inconclusive (16). The *ABCB1* polymorphism which has received greatest study is the c.3435C>T polymorphism (rs1045642), which is associated with marked reduction in *ABCB1* expression for the homozygous T genotype (17). This variant usually occurs as a haplotype including the c.2677G>T/A, and/or c.1236C>T polymorphisms (18). Although the effect of this haplotype has been studied for its potential role in colchicine effectiveness in familial mediterranean fever among other diseases (19), it has not yet been considered as a possible contributor to the development of colchicine myopathy.

Here we present two cases of colchicine-induced myopathy, with a description of muscle MRI findings, muscle pathology, sequencing of genetic polymorphisms previously associated with altered colchicine metabolism, and transcript level quantification of *ABCB1* from muscle tissue.

## Methods

### Ethics Statement

All participants provided written informed consent for participation in research and for publication of this case series. Control muscle samples were taken from patients with other acquired muscle diseases at the time of clinical muscle biopsy procedures. The study was approved by the University of Calgary Conjoint Health Research Ethics Board (REB15-2763 and REB16-2196).

### Clinical Case Reviews

Two patients with colchicine myopathy were identified from the clinical practice of two of the authors (GP, HE). Both patients provided written informed consent for participation in research and for publication of this case series. Clinical records were reviewed, including laboratory investigations, MRI images, and muscle pathology findings.

Genotyping of *ABCB1* polymorphisms: Four polymorphisms in *ABCB1* were genotyped based on their prior association with altered colchicine metabolism (rs1128501, rs1128503, rs2032582, rs1045642) (17, 20). DNA was obtained from peripheral blood using QIAamp DNA blood mini kit (Qiagen), amplified using custom primers, and Taq DNA Polymerase (Qiagen), followed by Sanger sequencing using BigDye (Applied Biosystems) on an ABI 3730XL sequencer (Applied Biosystems). Genotypes were determined using Variant Analysis software (Thermo Fisher).

### ABCB1 Transcript Quantification

*ABCB1* transcript quantification was performed on frozen muscle tissue of both patients and four controls. Total RNA was extracted using the RNeasy Fibrous Tissue Mini Kit (Qiagen), followed by reverse transcription with iScript cDNA Synthesis Kit (Bio-Rad) and random hexamers. qPCR was performed using custom primers and Evagreen (Biotium) on a QX200 digital droplet quantitative PCR system (Bio-Rad). Three experimental replicates were performed and included in data analysis and *HPRT1* was used as a reference gene. A non-parametric Kruskal–Wallis test was performed using GraphPad Prism 7.0, resulting in overall significance (*p*-value 0.0312), with Dunn's multiple comparison test being used to compare normalized expression of *ABCB1* between controls and patients of interest.

## Results

### Case 1

A 68 years old man started therapy with colchicine for gout at 0.5 mg/day. His other medications included irbesartan, allopurinol, and rosuvastatin. He had a diagnosis of ankylosing spondylitis but was not receiving any active medical therapy for this condition. He had normal renal function. Two weeks after starting colchicine he developed subacute onset of proximal weakness primarily affecting his legs and causing difficulty with gait and rising from a chair. On presentation to hospital he had 2/5 hip flexor weakness, 3/5 hip extension, and 4/5 knee extensor/flexor weakness, with shoulder abduction and adduction 4/5 weakness. On sensory examination he had reduction of vibration sensory thresholds to his knees but otherwise normal proprioception and pinprick testing.

**Figure 1:**
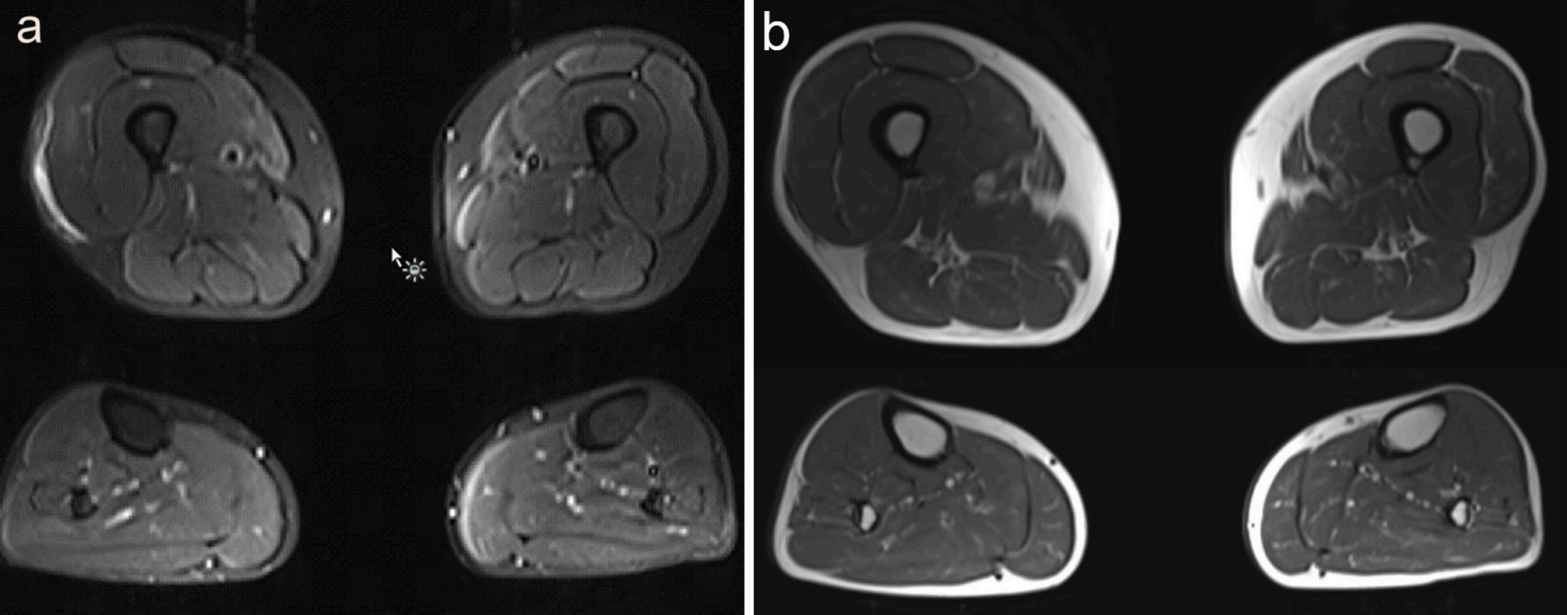
Muscle MRI findings in Case 1. **(a)** Axial images of STIR MRI sequences through the upper and lower legs show subtle signal change in the right medial thigh, which had been attributed to the patient's muscle biopsy a few days prior. Otherwise, no abnormality was identified on this study. **(b)** Axial images of T1 MRI sequences through the upper and lower legs show no abnormality.

**Figure 2:**
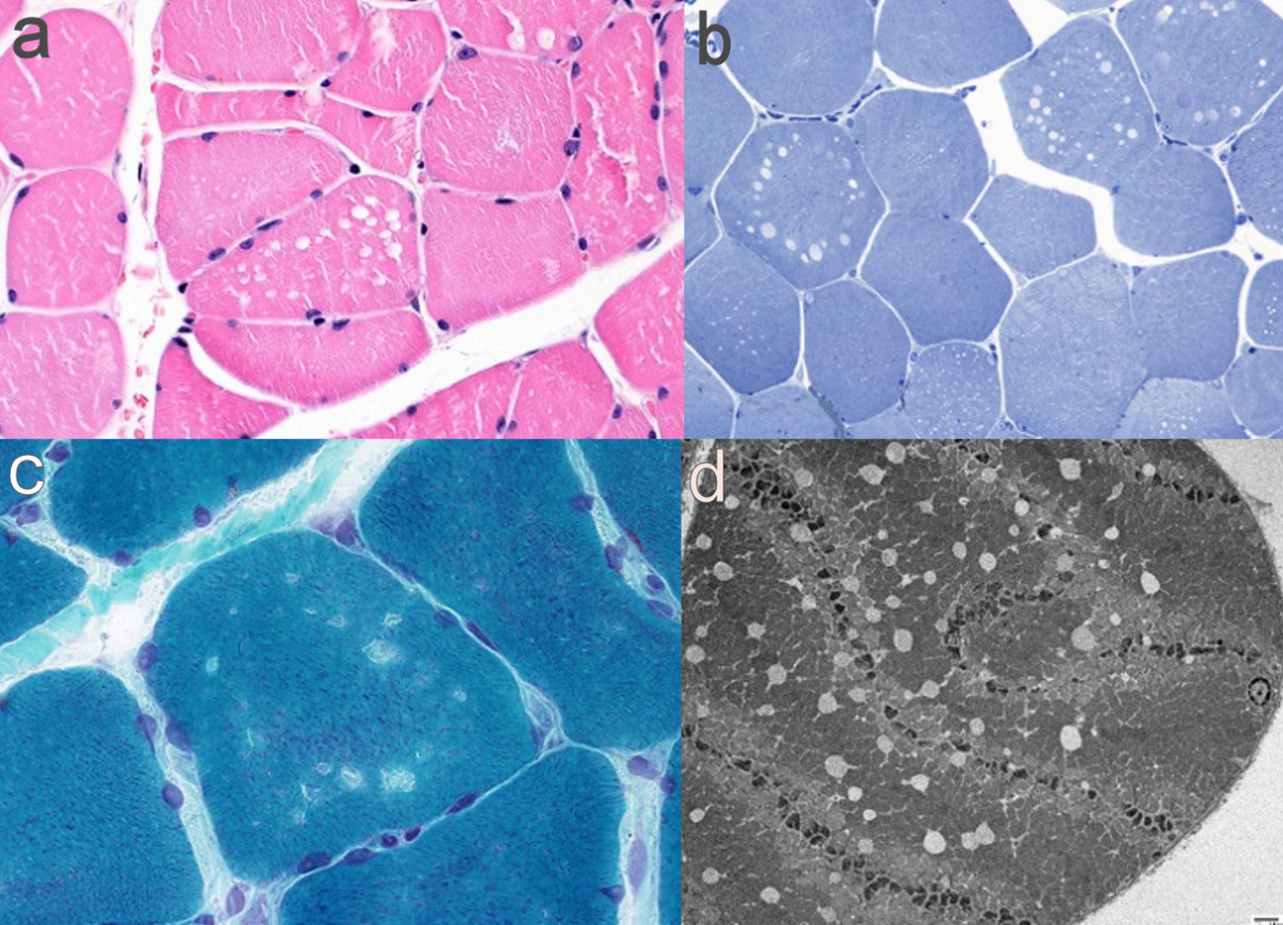
Muscle pathology findings in Case 1. **(a)** H&E-FFPE (400x) showing non-rimmed vacuoles; **(b)** Gomori-trichrome (600x) with non-rimmed vacuoles; **(c)** Toluidine blue semi thin sections (400x) with empty vacuoles; **(d)** electron micrograph (1,000x) showing empty vacuoles and Z-line disarray.

Investigations demonstrated an elevated CK to a maximum of 2,200 U/L (normal <350 U/L). EMG of the iliopsoas muscle revealed motor units with reduced amplitude and duration, and polyphasia, interpreted as representing myopathic changes. MRI (1.5T) of the leg muscles was acquired in coronal and axial planes with T1 and STIR sequences, from the top of the iliac crests to the ankles bilaterally, and was interpreted as unremarkable (Figure 1). Muscle biopsy showed a vacuolar myopathy confirming the clinical suspicion of colchicine myopathy (Figure 2).

Colchicine and rosuvastatin were withdrawn and CK levels normalized after 1 week. Weakness improved gradually toward normal during that time, although he still had 4/5 weakness in hip flexion. Follow-up after 2 months demonstrated complete resolution of clinical weakness, and his vibration sensory thresholds normalized (normal thresholds at medial malleoli bilaterally).

Eight months later the patient resumed treatment with rosuvastatin and has continued to use this agent without side effects after 2 years of follow-up.

### Case 2

**Figure 3:**
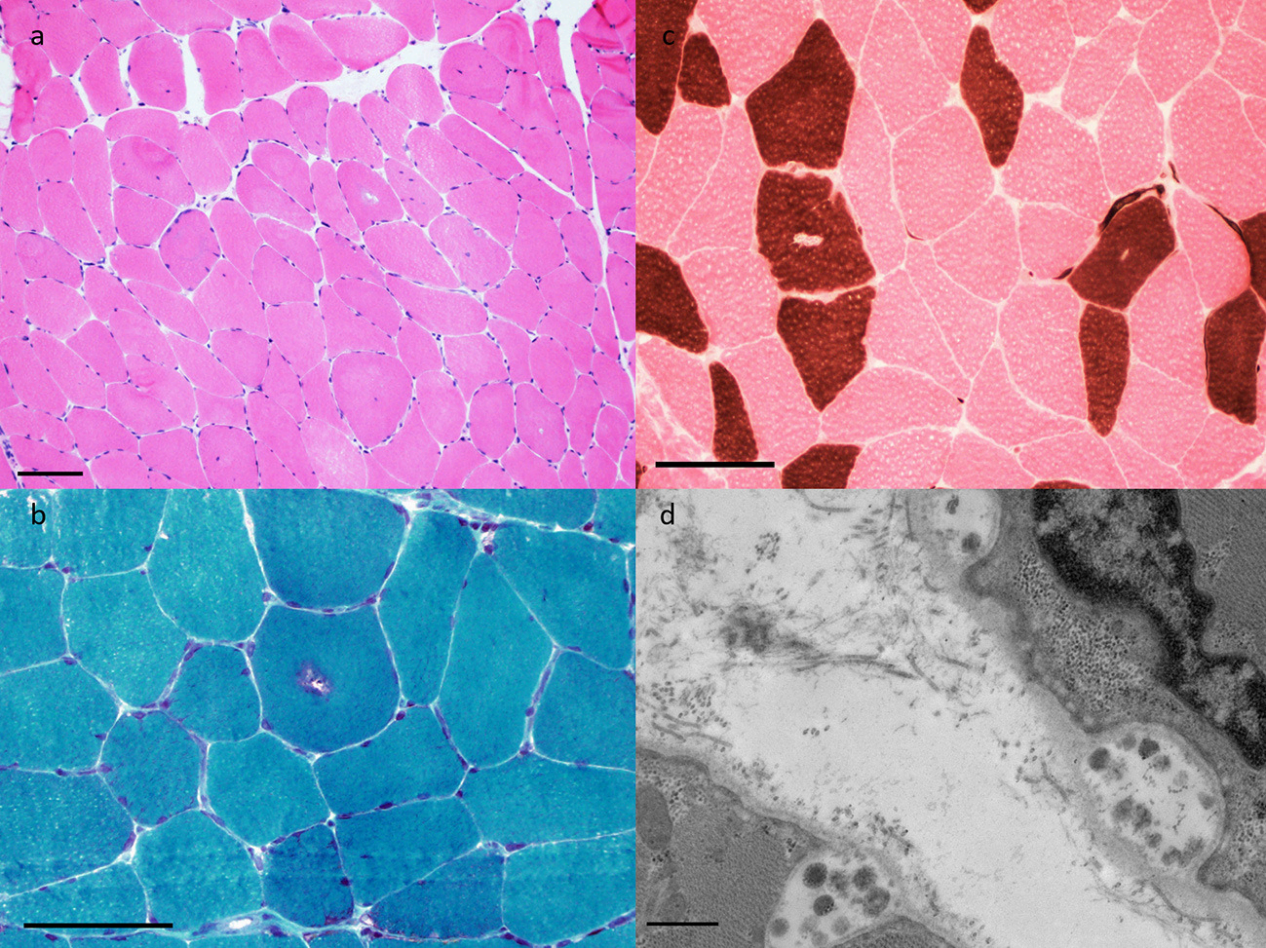
Muscle pathology findings in Case 2. **(a)** H&E-frozen (40X) showing rare rimmed vacuoles and increased internal nucleation, **(b)** Gomori trichrome (100X) highlights granular material of the typical rimmed vacuole, **(c)** ATPase pH4.3 (100X) shows vacuoles to have a predilection for Type I fibers, and **(d)** Electron microscopy (10,000X) demonstrates autophagic vacuoles.

The patient is a 72 years old man with a medical history of type 2 diabetes mellitus, chronic renal disease (GFR 29 ml/mn, secondary to above-mentioned diabetes), hypertension, and gout. He also had remote renal cell carcinoma, in remission following cryoablation. His medications included irbesartan, acarbose, repaglinide, allopurinol, rosuvastatin, and colchicine. He had three episodes of rhabdomyolysis over a 2 years period, separated by intervals of ~12 months, which presented clinically as proximal weakness, myalgias, and reduced mobility. Rhabdomyolysis was attributed to rosuvastatin, which was discontinued after the second episode, although he subsequently went on to have a reoccurance of rhabdomyolysis off statin therapy. His maximum CK levels were 4,021, 4,568, and 3,212 U/L, respectively in each of the three episodes. CK levels normalized in between episodes. After the third episode, he was referred to a neuromuscular specialist (HE). He had bilateral leg weakness causing difficulty with ambulation. EMG identified fibrillation potentials, positive sharp waves, with decreased amplitude, decreased duration and early recruitment upon muscle activation, interpreted as consistent with necrotizing myopathy. An MRI of the lumbar spine did not show any changes that would explain his weakness but did indicate degenerative disc disease particularly at the L3-5 levels. Muscle biopsy revealed vacuolar myopathy characterized by type I-specific central rimmed vacuoles that were reactive to acid phosphatase and immunoreactive to alpha-B-crystallin (Figure 3). This was interpreted as being consistent with colchicine myopathy. After discontinuation of colchicine the CK normalized over a period of 2 weeks, and the patient's weakness gradually improved such that he returned to ambulating without walking aids. After 1.5 years of follow-up he has not had further episodes of elevated CK.

### Genotyping of ABCB1 Polymorphisms

Case 1 was heterozygous for the polymorphisms rs1128503, rs2032582, rs1045642, which is potentially consistent with the heterozygous haplotype at positions c.1236/2677/3435. Case 2's sequencing showed a homozygous T genotype at the c.3435 position (reference sequence for these three SNPs). Both cases were homozygous reference sequence for the rs1128501 polymorphism.

### ABCB1 Transcript Quantification

**Figure 4:**
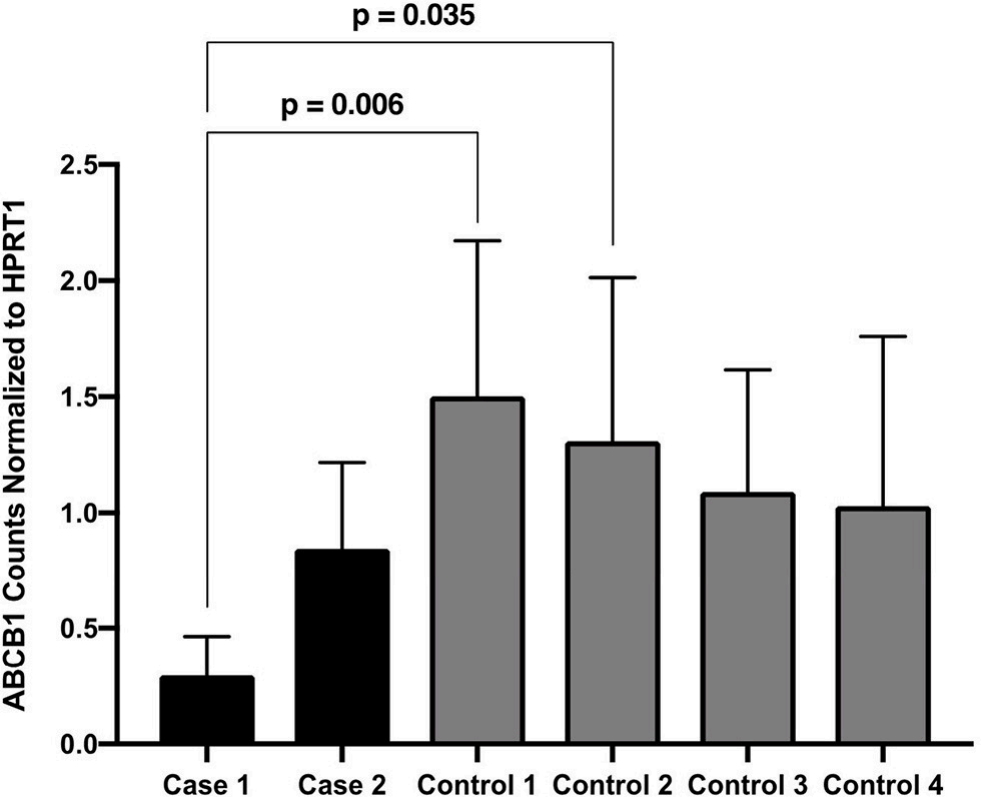
*ABCB1* Counts Normalized to *HPRT1*. Expression of *ABCB1* normalized to *HPRT1* for the two patients of interest and four disease controls, pooled from three assays performed in duplicate. Expression is quantified as the number of droplets containing *ABCB1* amplicons in the ddPCR assay. Dunn's multiple comparison tests significant *p*-values are indicated.

Descriptively, both cases had reduced *ABCB1* transcript levels compared with all four controls. Transcript levels for Case 1 was significantly reduced compared with two of the controls. Transcript levels from Case 2 did not differ significantly from controls (Figure 4).

## Discussion

We present two cases with colchicine myopathy, and demonstrate their clinical presentation, muscle pathology, muscle MRI, and targeted genetic analysis of *ABCB1*. This study contributes novel information in several ways. Firstly, this study demonstrates the first muscle MRI data on a patient with colchicine myopathy in the medical literature. Although this MRI study was normal, this is useful information from a clinical perspective, since muscle MRI is otherwise considered an extremely sensitive test for muscle injury and is increasingly used in diagnosis of hereditary, autoimmune, and acquired muscle pathology (21–23). Muscle MRI continues to be studied as a quantitative tool in muscle diseases (24), and is increasingly used in the characterization of unique clinical situations (25–27). The information presented from this case suggests that muscle biopsy is still the preferred test when colchicine-induced myopathy is suspected clinically, and also emphasizes that muscle MRI may not be sensitive for detection of all types of muscle pathology. Muscle MRI has been used in other drug-induced myopathies such as statin myopathy, which can show edema most commonly in the dorsal thigh and superficial dorsal leg muscles (28). Other examples of drug-induced myopathies are caused by use of antiretrovirals, corticosteroids, or cocaine among others, and general patterns of muscle MRI findings have been described (29). Further study of MRI findings in colchicine myopathy will be required to confirm whether any abnormalities can be identified with this imaging modality.

This study is also the only report in which genetic variation of *ABCB1* was considered as a possible contributing factor to colchicine myopathy. Our hypothesis was that genetic variation in *ABCB1* (such as the finding of a homozygous c.3435T allele) might be present in both patients, and provide some suggestion of a susceptibility for this rare adverse reaction. It is of potential interest that Case 1 was heterozygous and Case 2 was homozygous for the T allele, but based on this small series we cannot draw any conclusions regarding variants in *ABCB1* and colchicine myopathy. These genotypes could have occurred by chance based on the distribution of alleles in the general population [global MAF of 0.498 for the T allele in Gnomad (30), accessed February 22, 2019]. Given our descriptive findings of reduced *ABCB1* transcript levels, future study of the c.3435 polymorphism or other genetic factors in a larger series of colchicine myopathy patients could be considered.

Transcript quantification of both cases compared with disease controls using RT-qPCR showed reduced *ABCB1* transcript levels although statistical significance was not achieved, probably relating to our small sample size. Future study will be required with larger sample sizes to determine whether other patients also have a reduction of *ABCB1* transcript levels. If so, this might allow a way to predict which patients are more susceptible to develop colchicine myopathy or other complications relating to colchicine, based on presumed lower *ABCB1* activity.

The clinical presentation of these cases demonstrated some interesting features. Case 1 developed a painless proximal myopathy with neuropathy after 2 weeks of therapy, which is somewhat atypical for a patient without renal failure (6). In Case 2, there were repeated episodes of rhabdomyolysis as the condition had been attributed to his use of statins, and curiously his CK levels temporarily normalized after discontinuation of statins despite continuing to use colchicine. In both cases, the patients were on both rosuvastatin and colchicine—this raises the possibility that statins may have contributed to the development of colchicine myopathy, which is also supported from prior studies (31). However, the association of statin use with colchicine myopathy is still uncertain (32), despite what appears to be a high level of biological plausibility for this interaction from prior study (33). Further study of the factors that cause this rare reaction to colchicine will improve clinicians' ability to predict which patients will be at greatest risk. This report provides preliminary data that will hopefully be of interest in future studies of muscle MRI and pharmacogenetic interactions for colchicine and myopathy.

## Data Availability

Anonymized laboratory data will be made available upon request by any qualified investigator.

## Ethics Statement

This study was carried out in accordance with the recommendations of the Conjoint Health Research Ethics Board of the University of Calgary, with written informed consent from all subjects. All subjects gave written informed consent in accordance with the Declaration of Helsinki. The protocol was approved by the Conjoint Health Research Ethics Board.

## Author Contributions

MG: laboratory data acquisition and interpretation and drafting of manuscript. AN and DN: muscle pathology data acquisition and interpretation. KM: laboratory data acquisition and interpretation and editing of manuscript for intellectual content. HE and SC: interpretation of clinical data and editing of manuscript for intellectual content. GP: drafting of manuscript, interpretation of clinical and laboratory data, supervision of study.

### Conflict of Interest Statement

The authors declare that the research was conducted in the absence of any commercial or financial relationships that could be construed as a potential conflict of interest.
